# The Role of Interleukin-37 in the Pathogenesis of Allergic Diseases

**DOI:** 10.32607/20758251-2019-11-4-54-64

**Published:** 2019

**Authors:** I. P. Shilovskiy, M. E. Dyneva, O. M. Kurbacheva, D. A. Kudlay, M. R. Khaitov

**Affiliations:** National Research Center – Institute of Immunology Federal Medical-Biological Agency of Russia, Moscow, 115522 Russia

**Keywords:** IL-37, bronchial asthma, anti-inflammatory cytokines, pro-inflammatory cytokines, gene expression

## Abstract

Cytokines of the interleukin-1 (IL-1) family play an important role in the realization of the protective functions of innate immunity and are the key mediators involved in the pathogenesis of a wide range of diseases, including various manifestations of allergy. The IL-1 family includes more than 11 members. However, the functions of many of them remain to be elucidated. Recently, new members of the IL-1 family have been discovered. In 2000, several independent research groups reported the discovery of a new interleukin of this family, which was named IL-37, or IL-1F7 (according to the new nomenclature). IL-37 was assigned to the IL-1 family based on its structural similarity with other members of this family. The study of its biological properties showed that its activity changes in inflammatory diseases, such as rheumatoid arthritis, psoriasis, as well as allergic diseases (allergic rhinitis, bronchial asthma, and atopic dermatitis). However, unlike most members of the IL-1 family, IL-37 acts as a negative regulator of inflammation. Activation of IL-37 suppresses inflammation, resulting in the suppression of inflammatory cytokines and chemokines, which in turn prevents infiltration of pro-inflammatory cells, mainly eosinophils and neutrophils. The exact molecular and cellular mechanisms of the anti-inflammatory effect of IL-37 in the development of allergic diseases (AD) have not been fully studied. This review summarizes and analyzes the accumulated experimental data on the role of IL-37 in the pathogenesis of AD, such as allergic rhinitis, bronchial asthma, and atopic dermatitis.

## INTRODUCTION

The development of allergic diseases (ADs), such as bronchial asthma, allergic rhinitis, and atopic dermatitis, is influenced by many factors, including genetic predisposition [1], as well as exposure to allergens, infections, and other negative environmental factors. In certain regions of the world – for instance in EU countries – the incidence of ADs reaches 30%, with predicted growth to 50% expected in the next 15 years [2, 3]. Bronchial asthma (BA) is a heterogeneous disease; it is usually characterized by a chronic inflammation of the respiratory tract [2]. A distinctive feature of allergic BA (aBA), which accounts for about 70–80% of all cases of the disease, is an increased level of allergen-specific IgE antibodies in the serum [4, 5] and high eosinophil count in the blood, mucous membranes of the respiratory tract. and bronchoalveolar lavage (BAL) [6]. Allergic rhinitis (AR), which is characterized by chronic inflammation in the upper respiratory tract [7], can significantly reduce a patient’s quality of life by affecting his/her sleep and performance status [2]. Atopic dermatitis (AtD) is a multifactorial inflammatory skin disease which may be partly due to genetically caused impairment of the skin barrier function [8, 9]. According to various studies, AtD developed in childhood persists in adult age in 40–60% of cases [10].

Thus, taking into account the significant prevalence of ADs, the development of new methods of treatment and prevention remains a relevant biomedical issue. However, finding new methods of therapy is impossible without understanding the molecular mechanisms of the disease pathogenesis.

Based on current knowledge [11-13], two stages can be distinguished in the development of AD: the sensitization stage, which is accompanied by the occurrence of hypersensitivity to the allergen, and the effector stage, which is accompanied by inflammation, tissue injury, and remodeling (bronchi in case of BA, skin in AtD and nasal mucosa in AR). During the sensitization stage, an initial encounter with an allergen, which enters the body through the damaged epithelium and is presented by MHC-II molecules on antigen-presenting cells (APCs), occurs. APCs migrate to the regional lymph nodes and trigger polarization of naive Th0 cells to Th2 cells, which produce the cytokines IL-4, IL-5, IL-9, and IL-13, which are responsible for the main manifestations of ADs [14]. This process also promotes the differentiation of B cells into antibody-producing plasma cells. Under the action of IL-4 and IL-13, B cells switch from the synthesis of IgM antibodies to the synthesis of IgE antibodies, which are mainly responsible for the mediation of the subsequent allergic reactions of the body [11, 12].

During the effector stage, IgE-class antibodies interact with mast cells and basophils through the FceRI and FceRII receptors. At a second encounter with an allergen, (the mast cell)??? interacts with surface IgE antibodies, resulting in cell degranulation and the release of pro-inflammatory mediators. The mediators, in turn, recruit pro-inflammatory cells, cause vasodilation, promote microthrombi formation with local tissue damage, and also exert the spasmogenic effect that leads to the contraction of smooth muscle, such as bronchi in BA. In parallel, T2 cells penetrate via chemokine receptors from blood vessels to the inflammation area, where they are activated by the allergen and produce IL-4, IL-5, IL-9, and IL-13. The cytokines IL-4, IL-9, and IL-13 contribute to the overproduction of mucus by bronchial epithelium (in BA) or nasal mucosa (in AR). IL-5 promotes recruitment of eosinophils to the inflammation area. and their activation. Eosinophils, in turn, release the mediators of inflammation during degranulation, which results in damage to surrounding tissues [15-17].

To date, the role of Th2 cells and the cytokines produced by them in the development of ADs has been studied comprehensively (see reviews [18-20]). Meanwhile, there is data on the participation of some recently discovered cytokines in the development of AD. IL-33, a member of the IL-1 family, has been shown to be involved in the development of AD. Secreted by epithelial cells, IL-33 activates innate lymphoid cells 2 (ILC2), which produce significant amounts of IL-5 and IL-13, thereby enhancing the pro-allergic Th2 immune response (see reviews [21-23]). There are reports in scientific literature on the participation of IL-37, another recently discovered representative of the IL-1 family, in the pathogenesis of ADs. The current review is devoted to the role of this IL in the development of ADs.

## THE HISTORY OF THE DISCOVERY AND THE MOLECULAR AND GENETIC CHARACTERISTICS OF IL-37

Interleukin-37 (IL-37) belongs to the IL-1 family, which includes 10 other cytokines: IL-1α, IL-1β, IL- 1Rα, IL-18, IL-36α, IL-36Rα, IL-36β, IL-36γ, IL-38, and IL-33. IL-37 was discovered in 2000, when three research groups independently described five mRNA transcripts of this cytokine using *in silico *methods [24, 25, 26]. The study of the biological function of the *IL-37 *gene was significantly complicated by the fact that the gene is absent in mice; for this reason, generation of IL-37-defectient mice and subsequent comparison with wild-type mice carrying functional IL-37 was not possible [27]. Unlike in humans, IL-37 is absent in chimpanzees, although the functional cytokine gene has been identified in other primates [28].

*IL-37 *is located on chromosome 2q12-13, a locus containing the genes of most IL-1 family cytokines [29]. In mice, the *IL-1 *gene cluster is also located on chromosome 2 [30-32]. Both loci – human and mouse – are quite similar, with the exception of the region encoding *IL-37*, which is absent in mice [27]. At the same time, in primates, for instance, in gorillas, the *IL-37 *gene is located on chromosome 2 [28].

**Fig. 1:**
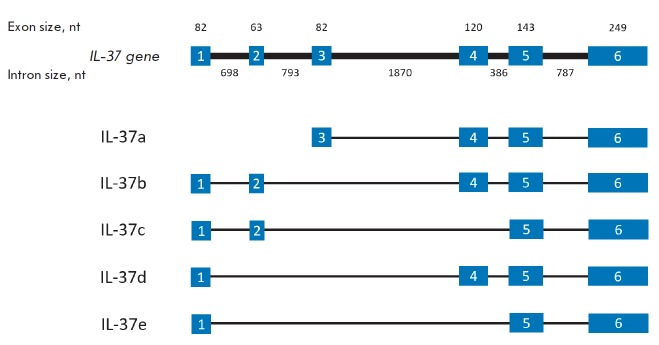
The structure of the gene *IL-37 *and its five alternative transcripts

The size of human *IL-37 *is 3,617 bp, and its mRNA undergoes alternative splicing, resulting in five different isoforms of IL-37: a–e *(Fig. 1)*. The isoforms a, b, and d contain the exons 4, 5, and 6. It would appear that the biological functions of *IL-37 *are associated with these exons [33].

IL-37a has a unique N-terminal sequence encoded by exon 3, which is the start exon for this isoform [31]. Axon 3 is absent in four other IL-37 isoforms, and translation of the protein starts at exon 1. Exons 4–6 encode the putative 12 β-folds, which then form the β-trefoil fold, a structure typical of all members of the IL-1 family [34].

The IL-37b isoform is the best-characterized; it consists of 218 amino acid residues. The N-terminal sequence encoded by the first two exons presents a pro-domain, which is cleaved during cytokine maturation. Exons 4–6 play the same role as in isoform IL-37a. Thus, one can assume that both the IL-37b and IL-37a isoforms have biological significance [33].

IL-37c isoform differs from IL-37b: it lacks exon 4, which does not allow IL-37c to form the typical β-trefoil structure during folding. Hence, we can assume that it lacks any biological function. The same is true for the IL-37e isoform, which also does not contain exon 4. Unlike for IL-37b, IL-37d lacks exon 2. Hence, it can form β-trefoils and also serve as a functional cytokine form [33].

Cytokines of the IL-1 family are synthesized as precursor molecules containing a pro-peptide domain. It has been established that caspase-1 is the main enzyme necessary for the processing of precursor molecules into mature cytokine forms and their subsequent secretion [35]. IL-37b is also synthesized as a precursor protein and processed into mature form after cell stimulation (for example, with LPS) [36]. The caspase-1 cleavage site is located in the sequence encoded by exon 1. Therefore, the isoforms b, c, d, e carrying exon 1 also contain a caspase-1 cleavage site. Isoform IL-37a does not contain exon 1 but has a caspase-1 cleavage site, a unique sequence located in exon 3.

Caspase-1 performs the most effective protein cleavage, while caspase-4 acts much slower; and other caspases do not show enzymatic activity against IL-37 [37]. Unlike for IL-33, the secretion of IL-37 is not associated with cell death. Apparently, processing of IL-37 by caspases (and/or other enzymes) is not necessary for its subsequent secretion, since both the processed form of IL-37 and its predecessor were detected in the extracellular space after activation [38]. However, it should be noted that in the case of IL-37, as well as some representatives of the IL-1 family (IL-1β and IL-33), both the processed form and its precursor possess biological activity [37]. Moreover, there is an assumption that unknown proteases can process the secreted mature form of IL-37 in the extracellular space and increase its activity [27]. It has been shown that the recombinant processed protein IL-37 (46–218 aa) lacking 45 amino acid residues at the N-terminus exhibits 20–30 times greater biological activity than the unprocessed protein [39].

Different isoforms of IL-37 were found in various tissues and organs; in some organs, only one isoform is expressed. For instance, only IL-37a is expressed in the brain, the expression of IL-37b is specific to kidneys, and IL-37c is expressed in the heart. Two isoforms, IL-37d and IL-37e, are expressed exclusively in the bone marrow and testes [25, 26]. Mature IL-37 and its proform are secreted by activated macrophages, dendritic cells (DC), and peripheral blood mononuclear cells (PBMCs) [40]. IL-37, which is secreted by these cells, exerts its biological effects via a unique receptor complex.

## IL-37 RECEPTOR COMPLEX AND SIGNALING PATHWAYS

**Fig. 2:**
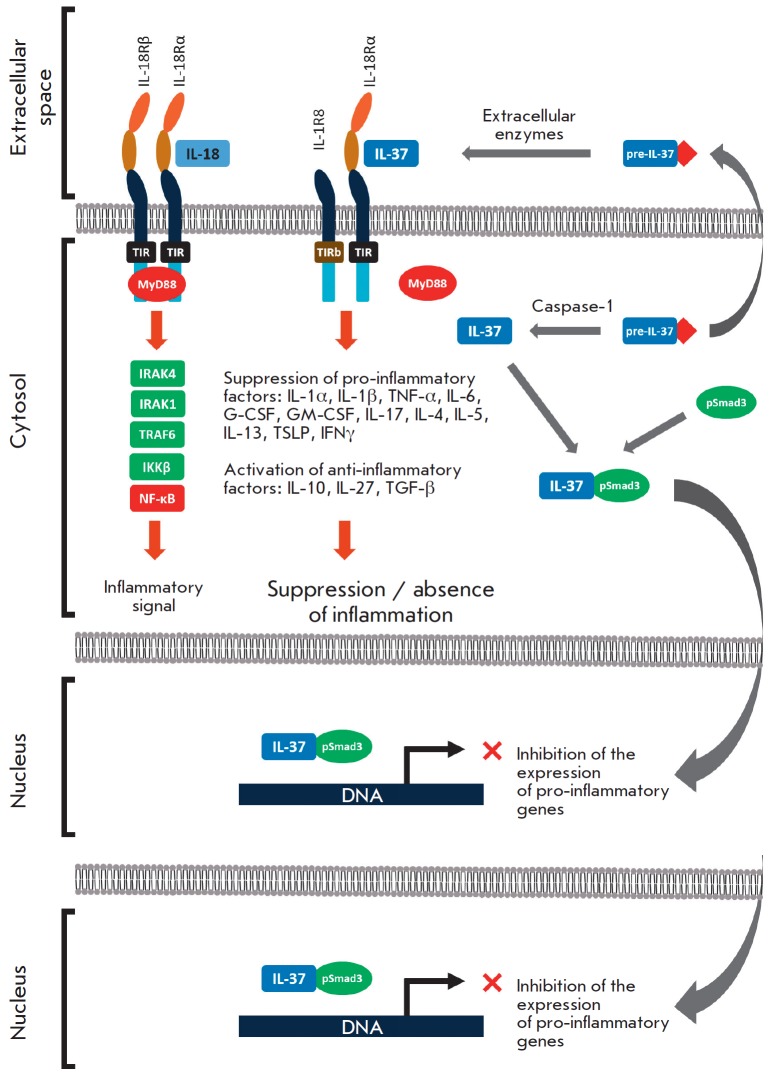
Mechanisms of the anti-inflammatory effects of IL-37. IL-18 exerts its pro-inflammatory effects through a receptor complex consisting of the IL-18Rα and IL-18Rβ chains. The TIR domains come together and further bind to the MyD88 factor inducing the pro-inflammatory effect. IL-37 is synthesized as a precursor (pre-IL-37) which is capable of secreting into the extracellular space, where it is processed to a mature form through an unidentified mechanism. Mature IL-37 binds to the chains IL-18Rα and IL-1R8 (instead of IL-18Rβ); at the same time, the IL-1R8 chain carries the mutant TIRb domain (instead of functional TIR), which does not allow realization of the MyD88-mediated inflammatory effect [21].The precursor of IL-37 is also capable of being processed intracellularly into mature form by Caspase-1. In the cytosol, IL-37 binds to the phosphorylated form of the Smad3 factor (pSmad3). Apparently, The IL-37 / Smad3 complex is able to translocate into the nucleus and inhibit the transcription of pro-inflammatory genes (the ability of the complex to bind to DNA has not been confirmed yet)

The IL-37 receptor complex is similar to the IL-18 receptor, another representative of the IL-1 family. IL-18 is one of the key pro-inflammatory cytokines acting as a pathogenetic factor in a number of diseases [41, 42]. The IL-18 receptor complex consists of two chains: α (IL-18Rα) and β (IL-18Rβ), each of which has a TIR domain [43]. During the formation of the IL-18Rα/IL- 18/IL-18Rβ complex, the TIR domains come together, after which the MyD88 factor binds to them and induces the pro-inflammatory effect *(Fig. 2)* [27].

IL-37 can also bind to the IL-18Rα chain; after knockout of this chain, IL-37 could not exert an anti-inflammatory effect [44]. Therefore, a hypothesis has been proposed that IL-37b is a competitive inhibitor of IL-18 and that IL-37b suppresses the inflammatory effect of this cytokine. However, this assumption was not confirmed [37, 45], after which another hypothesis was proposed: in addition to IL-18Rα, IL-37 can also bind to some receptor other than IL-18Rβ, which ultimately results in the activation of the anti-inflammatory pathway. It was soon established that an additional receptor for IL-37 is IL-1R8 (SIGIRR). It was shown that the IL- 37–IL-1R8–IL-18Rα complex is assembled on the cell surface, and that its presence is necessary for further triggering of an anti-inflammatory response [46]. The IL-1R8 receptor consists of only one extracellular Ig-like domain with a long "tail" immersed in the cytoplasm and containing a mutant TIR domain [47]. The involvement of IL-1R8 in the anti-inflammatory action of IL-37 has been demonstrated in experiments on mice defective in the gene for this chain. No reduction in inflammation was noted in IL-1R8 knockout mice in response to IL-37 administration (*Fig. 2*) [44, 48]. These facts indicate that IL-1R8 and IL-18Rα are required for the manifestation of the anti-inflammatory activity of IL-37.

In addition, IL-37 is capable of exerting an anti-inflammatory effect via the IL-1R8/IL-18R-independent pathway [36]. Studies of the human lung cancer cell line A549 demonstrated that A549 cells are less sensitive to inflammatory factors when IL-37 is associated with Smad3. At the same time, inhibition of Smad3 was shown to increase the production of inflammatory cytokines. *In vivo *experiments performed on IL-37tg mice revealed that the likelihood of LPS-induced pneumonia was increased after Smad3 suppression. However, the exact mechanism of interaction between IL-37 and Smad3 remains unclear. It is believed that the C-terminal domain of IL-37 binds to Smad3, undergoes phosphorylation and then enters the nucleus, where it inhibits the expression of pro-inflammatory genes *(Fig. 2)* [49].

Thus, IL-37 exhibits anti-inflammatory properties in extracellular and intracellular conditions. After intracellular synthesis, a portion of the precursor protein is processed by caspase-1 and then performs negative regulation of pro-inflammatory genes through the Smad3 pathway. Another portion of the IL-37 precursor protein is secreted into the extracellular space, where it is further processed and exerts an anti-inflammatory effect by competitively inhibiting the pro-inflammatory IL-18 and activating the anti-inflammatory signaling pathway via the IL-1R8 and IL-18Rα receptors.

## BIOLOGICAL EFFECTS OF IL-37

Most of the research, which has included *in vitro *and *in vivo *experiments, has studied the IL-37b isoform, which is of maximum size among all isoforms (218 amino acid residues). Mice lack IL-37 but have a functional receptor complex capable of binding human IL-37 [27, 39, 48]. For this reason, the biological role of this cytokine was studied not only using a cell culture, but also in laboratory mice.

In *in vitro *studies, recombinant IL-37b reduced the production of the pro-inflammatory cytokines IL-1β, IL-6, and TNF-α by human M1 macrophages after their stimulation with LPS [39]. Moreover, inhibition of IL-37 by monoclonal antibodies has the opposite effect [50]. In addition, IL-37 reduces the activation of pro-inflammatory cells, neutrophils, and prevents their migration along the chemokine gradient [51, 52]. Administration of IL-37 decreases IL-1β expression in mouse alveolar macrophages [48]. Furthermore, introduction of recombinant IL-37 inhibits the proliferation of Th17 cells in mice [53]. Thus, all this data indicates a pronounced anti-inflammatory activity of IL-37 against epithelial cells, macrophages, neutrophils, and blood mononuclear cells.

The biological role of IL-37 was studied *in vivo *in so-called IL-37tg mice with IL-37b gene insertion [49]. LPS was administered to these mice, after which the production of a number of pro-inflammatory and anti-inflammatory factors was evaluated. It was shown that LPS did not increase the level of the pro-inflammatory cytokines IL-6, IL-1β, IL-17, IFNγ,etc. both in wild-type and IL-37tg mice, while the level of anti-inflammatory cytokines, such as IL-10, increased both in transgenic and wild-type mice [36].

The role of IL-37 was also studied in a model of colitis induced by sodium dextran sulfate in mice. The severity of the intestinal inflammation was shown to be significantly lower in IL-37tg mice: infiltration of the colon by all types of leukocytes and production of inflammatory cytokines (IL-1β, IL-17, TNF-α) decreased, while production of anti-inflammatory IL-10 increased. Adoptive transfer of bone marrow cells from IL-37tg mice to wild-type mice led to a significant reduction in signs of experimental colitis. This indicates the anti-inflammatory effect of myeloid cells expressing IL-37 [49] (see review [27]).

The anti-inflammatory role of IL-37 was also confirmed in other models: in an experimental model of ischemic injury [54], acute renal ischemia [55], regional spinal cord injury [56], obesity, and type 2 diabetes in mice [57, 58] (see review [27]).

A number of authors attribute such an anti-inflammatory activity of IL-37 to its ability to attenuate the presentation of antigens and thereby suppress T cell activation. This assumption is supported by the fact that DC isolated from IL-37tg mice had a reduced level of CD40 and MHC class II molecules [59]. In addition, IL-37 increases the level of T regulatory cells, which suppress inflammation via secretion of the anti-inflammatory factor TGF-β [49, 60].

However, it still remains unclear how IL-37 exerts its impact: either via its intracellular form or by binding the extracellular IL-37 to its cell surface receptor. By using antibodies that neutralize extracellular IL-37, it was shown that this cytokine exhibits extracellular activity in some cases, since its neutralization in IL-37tg mice increased the level of pro-inflammatory IL-6 in the serum [38]. In other studies, on the contrary, neutralization of IL-37 in mouse macrophages transfected with the corresponding transgene did not affect their production of IL-6. This fact indicates that, in this type of cells, IL-37 functions more likely via intracellular mechanisms [38]. Injection of mature IL-37b or its precursor into human M1 macrophages suppressed the LPS-induced expression of IL-1β, IL-6, and TNF-α, but the effect was practically absent in M2 macrophages, DCs, and PBMCs [38, 39]. However, introduction of IL-37 into PBMCs of patients with rheumatoid arthritis reduced the expression of pro-inflammatory cytokines [53].

Thus, we can conclude that, in general, IL-37 is a negative regulator of the inflammatory process, apparently due to a decrease in the expression of the main pro-inflammatory cytokines, inhibition of DC maturation and their presentation of antigen, as well as due to the induction of T-regulatory cells and anti-inflammatory cytokines by IL-37.

## EXPERIMENTAL DATA ON THE ROLE OF IL-37 IN THE PATHOGENESIS OF ALLERGIC DISEASES

**Clinical cases**

The first data on the possible involvement of IL-37 in the pathogenesis of ADs appeared after an increase in the expression level of the largest isoform, IL-37b, was detected in the serum of a patient with AtD by using ELISA. Moreover, an increase in IL-37b concentration was directly correlated to the severity of disease symptoms. A study of the local gene expression in skin biopsy material by immunohistochemistry revealed an increase in the IL-37 level in epidermal keratinocytes and some stromal cells of the dermis, but not in lymphocytes infiltrating the skin tissue. Thus, IL-37 was induced both locally and systemically in patients with AtD, which may be due to the activity of another member of the IL-1 family, namely IL-18, in the skin, which is activated through TLR signaling in response to *Staphylococcus aureus *infection, a pathogen that is often present in large quantities on the skin of AtD patients. Considering that IL-18 is a pro-inflammatory cytokine, the simultaneous increase in the expression of anti-inflammatory IL-37 can be explained by a compensatory response of the body to excessive inflammation in AtD (*Table 1*) [61].

**Table 1:** The role of IL-37 in allergic diseases. Clinical cases

Pathology	Study design	Detection method	Result	Reference
AtD	55 adult patients with moderate and severe AtD	Evaluation of IL-37b in sera samples using ELISA, evaluation of local expression in skin biopsy material by immunohistochemistry.	Serum IL-37 levels are significantly higher in patients with AtD. IL-37 level positively correlated with the severity of AtD symptoms.	[61]
AR	40 children with AR (among them 10 with BA)	Evaluation of IL-37b in sera samples and nasal lavage using ELISA	The level of IL-37b was decreased in serum and nasal lavage in AR. The level of nasal Th2 cytokine was negatively correlated with local expression of IL-37b. Blood levels of ECP, IgE, and eosinophilia were negatively correlated with the level of serum IL-37b. Intranasal administration of a glucocorticosteroid drug led to the induction of IL-37b and a decrease in AR symptoms	[62]
aBA	21 children with aBA	Evaluation of IL-37 in the supernatants of stimulated PBMCs using ELISA	Production of IL-37 by PBMC-stimulated cells is significantly reduced in children with aBA.	[50]
aBA and nBA	92 children, among them 74 with aBA and 18 with nBA	The expression level of IL-37 in stimulated PBMCs was evaluated by RT-PCR	Patients with nBA have an increased level of IL-37, increased number of neutrophils in the blood, and increased level of pro-inflammatory cytokines IL-1β and IL-17.	[64]
BA	40 children with mild and moderate BA	The expression level of IL-37 in serum and sputum was evaluated using ELISA and RT-PCR	The expression of IL-37 mRNA in sputum and its serum level are significantly decreased in BA patients. Suppressed production of pro-inflammatory cytokines TNFα, IL-1β and IL-6 was observed in sputum cells cultured with rIL- 37 after cell stimulation with LPS. Stimulation of CD4+ T cells of the sputum in the presence of IL-37 decreased the production of IL-17.	[65]
BA	40 children with mild to moderate BA	Incubation of sputum cells in the presence of IL-37	The production of TSLP by sputum epithelial cells was significantly decreased in the presence of IL-37.	[66]
AR	32 adults with AR	Analysis of CD4+ and T cells isolated from PBMCs and cultured with rIL-37	After incubation with IL-37, the production of IL-17 and IL-4 by CD4+ T cells was significantly decreased in patients with AR.	[63]

Note:

AtD – atopic dermatitis;

AR – allergic rhinitis;

ELISA – enzyme-linked immunosorbent assay;

ECP – eosinophil cationic protein;

PBMCs – peripheral blood mononuclear cells;

BA – bronchial asthma;

aBA – allergic bronchial asthma;

nBA – non-allergic bronchial asthma;

RT-PCR – real-time polymerase chain reaction;

rIL-37 – recombinant interleukin-37.

On the contrary, a significant decrease in IL-37 activity in AR was found in a study published by Liu et al. [62]. In particular, a significant decrease in IL-37 concentration was observed both in the nasal lavage and systemic circulation of AR patients. Ten out of the 40 children with AR included in the study were diagnosed with BA. At the same time, no changes were observed in the systemic and local levels of IL-37. Despite the fact that AR is associated with dysregulation of the Th1/Th2 cytokine balance, a decrease in IL-37 activity in AR patients occurs due to the activation of Th2 cytokines ((IL-4, -5 and -13) and suppression of Th1 cytokines (IL-12 and IFNg). The severity of such manifestations of AR, as well as the level of specific IgE in the serum and eosinophilia negatively correlated with IL-37 activity. In a more detailed *in vitro *study [62], stimulation of PBMCs, which were obtained from AR patients, with recombinant IL-37 (rIL-37) suppressed the production of Th2 cytokines but had no effect on the production of Th1 cytokines and IL-10. In contrast, the production of IL-37 by PBMCs decreased significantly after stimulation with recombinant Th2 cytokines, did not change after stimulation with Th1 cytokines, and was activated in response to IL-10 [62]. Similar results were obtained by Li et al. [63]. They confirmed that the production of both IL-4 and IL-17 is suppressed in the presence of recombinant IL-37 in the cultures of T cells isolated from the PBMCs of AR patients. However, IL-37 did not affect the production of such cytokines as IL-1b, IL-6, and IL-10 by dendritic cells isolated from the PMBCs of the same volunteers and did not alter the expression of the co-stimulatory molecules CD80, CD40, HLA-DR, and CD86 on their surface. Moreover, the presence of IL-37 in the culture medium did not affect the ability of DC to activate the production of IL-4 and IL-17 by T cells. This suggests that IL-37 acts as a regulator of innate rather than adaptive immunity (*Table 1*) [63]. It is known that Eosinophils secreting the eosinophil cationic protein (ECP) and other pro-inflammatory factors are known to be actively involved in the damage to the respiratory tract epithelium. A dose-dependent decrease in ECP was observed in eosinophils isolated from the peripheral blood of children with AR and then treated with IL-37, which confirms the anti-inflammatory role of this cytokine. The use of nasal steroid agents is one of the most common approaches in the treatment of this disease. In this study, a four-week course of corticosteroids resulted in a twofold decrease in the severity of AR symptoms and a subsequent significant increase in IL-37. Thus, Liu et al. showed that the development of AR symptoms is associated with a decreased IL-37 activity, while restoration of IL-37 expression reduces the disease symptoms (*Table 1*) [62].

Changes in the expression of IL-37 were also studied in aBA, and a significant decrease in IL-37 production by stimulated PBMCs harvested from children with aBA was shown in comparison with healthy volunteers [50]. A reduced expression level of a number of genes of the innate immune system, including the gene encoding for IL-37, was also demonstrated in children with aBA [64]. This effect is ascribed to the activity of Treg cells, whose blood count is increased in children with aBA. Moreover, these cells were able to suppress IL-5, IL-13, and IFNg in experiments *in vitro*. In children with non-allergic BA (nBA), despite the increased number of Treg cells, a significant increase in the expression of IL-37, as well as the pro-inflammatory cytokines IL-1b and IL-17, is observed, which is associated with a different functional state of Treg cells in children with nBA. Unlike for Treg cells isolated from children with aBA, the Treg cells of children with nBA were unable to suppress the expression of pro-inflammatory cytokines in *in vitro *experiments (*Table 1*) [64].

More evidence of a decrease in IL-37 activity in children with BA has been obtained. A significant decrease in IL-37 expression was found both at the level of mRNA and protein in the serum, as well as in the sputum of children with controlled BA (40 children in total, about 70% of whom had allergic asthma) compared with healthy volunteers. In addition, cells isolated from the sputum of children with asthma and cultured in the presence of rIL-37 exhibited a decrease in the production of the pro-inflammatory cytokines IL-1b, IL-6, and TNF-α, which confirms the anti-inflammatory properties of this cytokine. Similarly, the production of IL-17 was decreased in the CD4^+^ T cells of the sputum in the presence of IL-37, which indicates the ability of IL-37 to exert an anti-inflammatory effect via its direct effect on cells (*Table 1*). This data indicates that IL-37 deficiency in BA contributes to inflammation in this pathology [65]. In a similar study, rIL-37 inhibited the production of another pro-inflammatory factor, TSLP, by epithelial cells isolated from the sputum of children with BA (*Table 1*) [66].

Summarizing the results of studies of the role of IL-37 in the pathogenesis of AD, we can state that this cytokine has pronounced anti-inflammatory properties, which are realized via its direct action on eosinophils, T cells, and epithelial cells. Most studies have demonstrated a decrease in both the systemic and local activities of IL-37 in such ADs as aBA and AR. Apparently, the low activity of IL-37 contributes to a more severe course of the Th2-mediated pathology. However, different results were obtained in a number of studies: for example, an increase in the systemic and local expressions of IL-37 was shown in AtD. A possible explanation for this difference might be related to the specificity of the AtD pathogenesis, in which Th2 cells play a crucial role at the early stage of the disease, while Th1 cells are involved in the late stage [67]. In addition, an increase in the systemic and local activities of IL-37 was also shown in nBA. Bronchial asthma is a heterogeneous disease; it can develop not only via the pro-allergic Th2-dependent pathway, which is associated with the infiltration of eosinophils in the lung tissue, but also via the Th17- dependent pathway, when other pro-inflammatory cells, namely neutrophils, are detected in the lungs. Considering the heterogeneity of the BA pathogenesis, it was suggested that IL-37 might play a different role in different BA endotypes. This assumption was confirmed by Raedler et al. [64], who observed a decrease in IL-37 production in children with aBA, while IL-37 expression was increased in nBA.

Such contradictory data on the changes in IL-37 activity indicate the heterogeneity of the molecular mechanisms of ADs. It is possible that the inclusion of patients with a more accurate phenotyping in such studies will allow us to better understand the biological role played by this interleukin.

**Animal studies**

Studies on laboratory animals allow a more detailed evaluation of the biological role of a factor, since there is a wider range of instrumental methods of molecular biology that are not available in clinical practice. Such methodological tools include the use of neutralizing monoclonal antibodies, generation of knockout mice, and the use of rIL-37.

It is known that mice lack the gene encoding for IL-37. However, a receptor complex capable of activating the intracellular signal upon interaction with human IL-37 is localized on their cell surface. In one of the first such studies [48], the effect of rIL-37 obtained in *E. coli *cells was studied using a model of pulmonary aspergillosis in mice. Mice were subjected to sensitization with *Aspergillus fumigatus *fungus parenterally with further intranasal administration of the same pathogen. A few hours prior to intranasal provocation, mice were injected intraperitoneally with rIL-37 in a wide range of doses, from 1000 to 1 ng/mouse. IL-37 at doses of 1000 and 100 ng/mouse prevented lung tissue damage, which manifested itself in suppressed lung infiltration by neutrophils, Th2 and Th17 cells, and in reduced bronchial remodeling, such as peribronchial collagen deposition and metaplasia of bronchial epithelium. IL-37 was shown to reduce the level of the pro-inflammatory cytokines IL-1β, IL-6, and IL-17A in lung tissue and to activate IL-10 [48]. Intranasal administration of recombinant human IL-37 at a dose of 1 μg/mouse decreased the level of the pro-inflammatory cytokines IL-6, IL-12, IL-4, IL-5, and IL-13 in the BAL of OVA-induced asthmatic mice. Furthermore, a decrease in the level of these interleukins resulted in attenuated symptoms of experimental BA: there was a significant reduction in lungs eosinophilia, signs of bronchial remodeling, as well as bronchial hyperreactivity. The biological effect of IL-37 in this BA model could be due to its ability to competitively bind the IL- 18Rα receptor for the pro-inflammatory IL-18. However, further experiments on animals with gene knockout of receptors IL-18Rα and SIGIRR demonstrated a loss of the positive effects of IL-37 upon inactivation of the abovementioned receptor chains. This suggests that IL-37 does not only act as a competitive inhibitor of IL-18 but also activates its own anti-inflammatory signals [50]. Similar results were obtained in a similar OVA-induced BA model in mice [68]. Intranasal administration of IL-37 at a dose of 1 μg significantly attenuated the manifestations of BA in mice. In particular, there was a decrease in bronchial hyperreactivity and pneumonia, which is ascribed to a suppression of the pro-inflammatory Th2 cytokines IL-4, IL-6, and IL-13 and activation of the Th1 cytokine IFNg (*Table 2*) [68].

**Table 2:** Virus titer (lg FFU/mL) after treatment of the influenza virus with KS-6469

Animal model/species	Experimental protocol	Result	Reference
Pulmonary aspergillosis C57BL/6 mice	Intraperitoneal administration of rIL-37 at doses of 1000, 100, 10 and 1 ng/mouse prior to infection	Decrease in the number of neutrophils in the BAL Suppression of NLRP inflammasome in the lungs Decreased IL-1β, IL-6, and IL-17A in the lung tissue Activation of IL-10 in the lung tissue Reduced signs of bronchial remodeling (collagenosis of lung tissue and metaplasia of bronchial epithelium) Suppression of lung infiltration with Th2/Th17 cells.	[48]
OVA-induced BA. C57BL/6 mice	Intranasal administration of rIL-37 at a dose of 1 μg/mouse 1 day prior to aerosol administration of OVA	Decreased eosinophil count in BAL and lung tissue Suppression of bronchial epithelium hyperplasia, mucus production and bronchial hyperreactivity Suppression of pro-inflammatory cytokines in BAL: IL-6, IL-12, IL-4, IL-5, and IL-13	[50]
HDM-induced AR BALB/c mice	Intranasal administration of rIL-37 at a dose of 1 μg/mouse in combination with nasal provocation with HDM	Threefold reduction in nasal hyperreactivity Decreased levels of allergen-specific antibodies of the IgE class Suppression of eosinophilic infiltration to the mucosa of the nasal cavity Suppression of the pro-inflammatory cytokines IL-4, IL-5, IL-13 and IL-17 in the nasal mucosa and activation of regulatory IL-10	[70]
OVA-induced BA BALB/c mice	Intranasal administration of rIL-37 at a dose of 1 μg/mouse in combination with allergen provocation	Decreased bronchial hyperreactivity and lung infiltration by pro-inflammatory cells (lymphocytes, neutrophils, and eosinophils) Inhibition of IL-4, IL-6, and IL-13 in lung tissue Decrease in proliferation and migration of respiratory smooth muscle cells and epithelial-mesenchymal transition	[68]
HDM-induced BA BALB/c mice	Intranasal administration of rIL-37 at a dose of 0.2 μg/mouse combined with sensitization or nasal provocation with HDM	Decrease in the number of eosinophils in BAL and lung tissue, decreased bronchial hyperreactivity IL-37 suppressed IL-4/13-induced production of CCL11 fibroblasts and respiratory smooth muscle cells	[69]

Note:

rIL-37 – recombinant interleukin-37;

BAL – bronchoalveolar lavage;

BA – bronchial asthma;

OVA – ovalbumin allergen;

HDM – house dust mite allergen.

A more detailed study of the molecular and cellular mechanisms of the anti-inflammatory effect of IL-37 was conducted by Lv J. et al. [69] in a mouse model of BA induced by a house dust mite (HDM) allergen. Unlike in the case of OVA-induced BA, the HDM model is closer to the clinical case in humans, since a clinically significant allergen is used. Aerosol exposure without intraperitoneal sensitization was used for the administration of HDM to mice at the stages of sensitization and provocation. Intranasal administration of rIL-37 at a dose of 0.2 μg/mouse did not suppress BA symptoms, while administration of IL-37 at the provocation stage significantly attenuated disease manifestations, such as eosinophilic pneumonia and bronchial hyperreactivity. It is noted that, unlike in the study by Lunding et al. [50], IL-37 did not affect the differentiation of Th2 cells in the lungs and did not suppress the production of IL-4, IL-5, IL-13, and IL-17A. Moreover, no IL-37 effect on the production of IgE antibodies was detected. These results indicate that IL-37 is incapable of inhibiting T cell activation. However, despite high levels of Th2 cytokines, a significant suppression of the CCL11

A similar anti-inflammatory effect of IL-37 was revealed in a mouse model of AR. Kim et al. [70] induced AR in mice by intraperitoneal injection of the HDM allergen, followed by intranasal provocation with the same allergen. As a result, the animals developed AR signs: an increased level of IgE, eosinophil infiltration of the nasal mucosa, and nasal hyperreactivity, which manifested itself in an increased frequency of sneezing. Intranasal administration of rIL-37 (1 μg/mouse) in combination with allergen provocation resulted in mitigated symptoms of the pathology. Apparently, the attenuation of AR manifestations in mice is associated with the ability of IL-37 to suppress the activity of the pro-inflammatory cytokines IL-4, IL-5, IL-13, and IL-17 and activate regulatory IL-10 (*Table 2*) [70].

## CONCLUSION

Almost 20 years have passed since the discovery of IL-37. During this time, a large body experimental evidence of the anti-inflammatory properties of this cytokine has been accumulated. Analysis of the data on the role of IL-37 in ADs confirmed the unique function of IL-37 as an anti-inflammatory agent, which is atypical of other representatives of the IL-1 family. The results achieved in studies using both clinical material obtained from AD patients and mouse AD models showed that activation of IL-37 leads to the suppression of pro-inflammatory Th2 cytokines (IL-4, IL-5 and IL-13), Th17 cytokine (IL-17A), chemokines, and transcription factors (CLL11, STAT3, NF-κB, etc.). Suppression of these cytokines and factors ultimately leads to an attenuated inflammation, which manifests itself as a decrease in the degree of infiltration of the target organs (nasal mucosa in AR and lung tissue in BA) by pro-inflammatory cells (neutrophils and eosinophils) and a decrease in pulmonary hyperreactivity. The extracellular form of IL-37 exerts its biological effect through the receptor complex consisting of IL-18Rα and IL-1R8 chains; the intracellular form of IL-37 is able to translocate into the nucleus and inhibit the expression of pro-inflammatory genes. The revealed positive effects of IL-37 allow us to offer it for consideration as a potential anti-inflammatory agent for cytokine therapy of ADs.
